# On the history of plasma treatment and comparison of microbiostatic efficacy of a historical high-frequency plasma device with two modern devices

**DOI:** 10.3205/dgkh000251

**Published:** 2015-06-02

**Authors:** Judith Napp, Georg Daeschlein, Matthias Napp, Sebastian von Podewils, Denis Gümbel, Romy Spitzmueller, Paolo Fornaciari, Peter Hinz, Michael Jünger

**Affiliations:** 1 Department of Dermatology, University Medicine, Greifswald, Germany; 2 Department of Trauma Surgery, University Medicine, Greifswald, Germany; 3 Clinic of Orthopaedic Surgery, HFR Fribourg - Hôpital cantonal, Fribourg, Switzerland

**Keywords:** plasma medicine, low temperature atmospheric pressure plasma, historic plasma apparatus, antimicrobial efficacy

## Abstract

**Background:** Cold atmospheric pressure plasma (CAP) with its many bioactive properties has defined a new medical field: the plasma medicine. However, in the related form of high-frequency therapy, CAP was even used briefly a century ago. The aim of this study was to review historic CAP treatments and to obtain data regarding the antimicrobial efficacy of a historical high-frequency plasma device.

**Methods:** First, historic literature regarding the history of CAP treatment was evaluated, because in the modern literature no data were available. Second, the susceptibility of 5 different bacterial wound isolates, cultured on agar, to a historic plasma source (violet wand [VW]) and two modern devices (atmospheric pressure plasma jet [APPJ] and Dielectric Barrier Discharge [DBD]) was analyzed . The obtained inhibition areas (IA) were compared.

**Results:** First, the most convenient popular historical electromedical treatments produced a so-called effluvia by using glass electrodes, related to today’s CAP. Second, all three tested plasma sources showed complete eradication of all tested microbial strains in the treated area. The “historical” cold VW plasma showed antimicrobial effects similar to those of modern APPJ and DBD regarding the diameter of the IA.

**Conclusion:** Some retrograde evidence may be deducted from this, especially for treatment of infectious diseases with historical plasma devices. The underlying technology may serve as model for construction of modern sucessive devices.

## Zusammenfassung

**Hintergrund:** Kaltes Atmosphärendruckplasma (CAP) hat durch seine mannigfaltigen bioaktiven Eigenschaften ein neues medizinisches Feld definiert: die Plasmamedizin. Allerdings wurde vor etwa 100 Jahren CAP in verwandter Form in der Hochfrequenztherapie genutzt. Zielsetzung dieser Studie war eine Übersicht über die historischen Plasmabehandlungen zu gewinnen und Daten bezüglich der antimikrobiellen Wirkung eines historischen Hochfrequenzapparats zu gewinnen.

**Methode:** Erstens wurde historische Literatur bezüglich CAP-Behandlungen ausgewertet, da aus dem heutigen Schrifttum keine Angaben gewonnen werden konnten. Zweitens wurde die Empfindlichkeit von fünf verschiedenen bakteriellen Wundisolaten auf Agar gegenüber einer historischen Plasmaquelle (violet wand [VW]) und zwei modernen Geräten (atmospheric pressure plasma jet [APPJ] und Dielectric Barrier Discharge [DBD]) ermittelt. Die erzielten Hemmhöfe wurde verglichen.

**Ergebnisse:** Die seinerzeit populärsten elektromedizinischen Anwendungen erzeugten durch Glaselektroden sogenannte Effluvien, die mit modernem CAP verwandt sind. Alle drei untersuchten Plasmaquellen zeigten eine vollständige Eradikation aller behandelter Isolate im plasmabehandelten Bereich. Die historische Plasmaquelle (VW) war dabei ähnlich wirksam wie die modernen Plasmaquellen.

**Schlussfolgerung:** In begrenztem Umfang kann retrograd ein Wirksamkeitsnachweis der historischen Plasmabehandlungen abgeleitet werden, insbesondere bei der Behandlung infektiöser Erkrankungen. Die zugrunde liegende Technologie könnte für die Entwicklung moderner Nachfolgegeräte genutzt werden.

## Introduction

Cold atmospheric pressure plasma (CAP) lays the foundation for the completely new medical field of plasma medicine thanks to its numerous bioactive properties [1], [2], [3]. At present in plasma medicine beside the two main fields of basic and applied research, the treatment of chronic wounds [4], [5], [6], [7], [8], [9], [10] and the eradication of different superficial cancer [11], the determination of the antimicrobial efficacy of CAP is an another important focus of plasma medicine [12], [13], [14], [15], [16], [17], [18], [19], [20], [21], [22], [23], [24], [25].

During the early decades of the last century, the application of high-frequency irradiation was recommended for different diseases. In this period many apparatus used produced spark effluvia via glass electrodes. This effluvia is a form of CAP, a fact mostly unknown to modern scientists in plasma medicine. High-frequency devices (e.g., the violet wand [VW]) were commonly sold for home-care medicine [26] until the early 1950s. Recently, the authors showed similar to modern plasma sources antimicrobial properties of VW generated plasma [27]. In short, modern CAP treatment seems a rediscovery rather than a new invention. 

**Figure 1:**
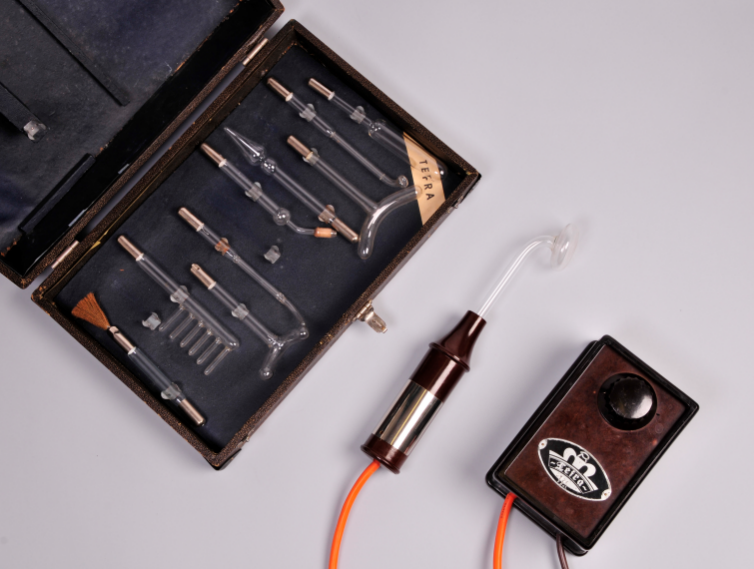
Violet wand (VW) plasma, left to right: assortment of electrodes, hand-held wand, and control unit

First, we performed a review of historic literature regarding electro medicine, as modern literature does not exist. Second, we tested a historical high-frequency device (Figure 1 (Fig. 1)) for its antimicrobial efficacy and compared its results with the results of two plasma devices based on different modern technologies. These plasma sources have proven marked antimicrobial activity *in vitro* [3], [28], [29], [30], [31] including efficacy against biofilms and the literature supports many other biomedical applications [32], [33], [34], [35], [36].

## Overwiew of historical plasma treatment

**Figure 2:**
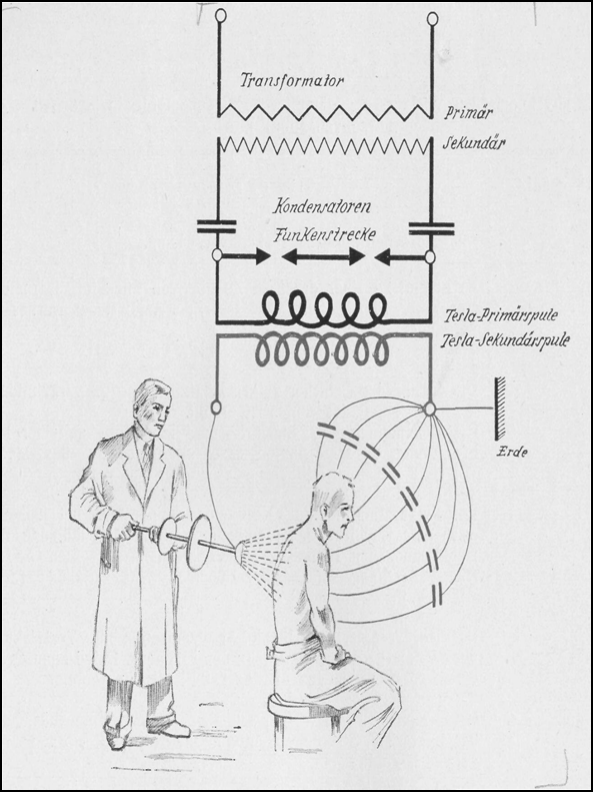
Electrical circuit and diagram of high-frequency treatment (skin touched by plasma spark filaments) (from: Holzer W. Physikalische Medizin in Diagnostik und Therapie. 5. und 6. erw. Aufl. Wien: Maudrich; 1947, XIV)

Electromedicine was a common medical practice in the early decades of the 20^th^ century and efficacy was claimed for a wide spectrum of diseases. Arsonvalization (Figure 2 (Fig. 2)) was one of the most convenient popular electromedical treatments, classified as high-frequency therapy, and had a lot in common with modern CAP treatment, at least in terms of bioactive properties. This historical device produced a so-called effluvia by using glass electrodes, related to today’s CAP using glass electrodes. While in the first applications, pure field effects were induced from a distance, the technical development of later therapeutic devices allowed direct body and skin contact with plasma discharges.

The French physiologist Jacques-Arsène d’Arsonval (1851–1940) [37] discovered the possibility of influencing the human body with high frequencies delivered by his apparatus with the help of extremely high transformation of electric tension. Technically, this had recently been made feasible by Nikola Tesla, who worked with extremely high-frequency currents at high voltage, creating impressive light phenomena which proved harmless to humans in the case of direct contact with the effusions.

In Germany, the devices were further developed for the caloric treatment of patients (diathermy) [38]. Rumpf developed a device which differed from the French ones by implementing a capacitively coupled electrode consisting of a Leydener bottle which was directly applied to the patient’s skin. This device can be considered the first plasma source in medicine to use a dielectric electrode and can be defined as directly related to the first plasma device in chemistry, which was invented by Siemens in 1888 to produce ozone.

The Leydener bottle used as an electrode was soon replaced by rubber (with ferrite inlay), and after industrial introduction of small hand-held devices, the waves were applied to the body and skin via vacuum, condenser, or brush electrodes producing glow discharge plasmas, brush lights, or spark effusions. The resulting discharge type mainly depended on voltage, the distance to the treated skin, the type of skin and soft tissue under treatment, the individual skin resistance, and the shape and construction of the chosen electrode. It was well known that the area of effective irradiation surpasses the visibly treated surface by far (about tenfold).

The plasma-skin interaction was described as “electric effluvia” in the form of glow or bunch discharge, the latter creating more intense skin irritations and erythema by secondary capillary dilation, leading to decreased arterial blood pressure, among other things [39], [40]. Depending on the disposition of the patient, erythema lasting for hours was reported [41]. At the cellular level, microscopic alterations such as karyorrhexis, pyknosis, leucocytic infiltrations and cellular micro-extravasates were described. These effects were discussed as potential effects caused by *de novo* generated proteins (“protein therapy”) [41] or “anionic” effects [42]. The claimed efficacy of historical plasma treatments was explained by chemical, mechanical, and optical changes, i.e. the chemical ones as splitting of electrons from N and O molecules, creating new molecules such as ozone, nitric and nitrous acid, the mechanical effect as the acceleration of air molecules by ions to form “ion wind” (explained with glowing wires beginning to oscillate), and the optical plasma effects as UV radiation [26].

Regarding safe use, the high frequency waves were known not to interfere with motoric and sensory nervous conduction, but they do cause narcosis at high doses in animals [42]. Furthermore, the strong skin irritation effect was known to stimulate the respiration (increase of the respiratory volume) [43] and antimicrobial efficacy against *E. coli*, *Salmonella typhi*, *C. diphtheriae*, and *M. tuberculosis*, was shown [44]. 

### Practical use

Arsonvalization was performed as local or “systemic” therapy. Local therapy was executed with skin electrodes, the latter indirectly with the help of large coils. The treatment was applied either in uni- or bipolar mode. The bipolar technique worked with the patient electrically connected to the grounded phase of the Tesla coil. In unipolar treatment, the circuit was closed via air capacity. A common treatment with glow discharge plasma (“effluvia therapy”) took 5–15 min. When spark effusion was desired for therapy, metal brushes were used. This treatment took 1–5 min (depending on tolerability) [26].

### Technique, power, and electrodes

The energy of arsonvalization was capacitively or inductively coupled to the body surface and tissues. Wave generation in the former was realized via spark gaps [26].

Most commonly, gas-filled vacuum electrodes were used, producing plasma glow discharges and creating vacuum discharges of different colors according to the gas employed [26]. 

### Medical indications and practice

Typical recommended indications were lichen ruber, cervical catarrh, arterial hypertension, eczema, pruritus, migraines/neuralgia, infectious diseases of the skin, and wounds [26], [38], [44], [45], [46], [47], [48], [49], [50]. Another indication in dermatology was the treatment of hemorrhoids, skin tumors (carcinomata), viral warts, furuncles and abcesses [26], [50], [51], [52]. Healing effects on tuberculosis were claimed repeatedly [50], [53], [54]. In dentistry, Henseler [55] described many procedures and indications including anesthesia before tooth extraction, but also antiseptic treatments and treatment of abcesses, stomatitis, and hyperesthesia. Tooth bleaching, gingival and pulpa anesthesia were also common [55]. Further treatments in dermatology were iontopheresis and the improvement of topical drug penetration [56] which is a promising perspective for modern plasma sources [57], [58], [59], [60], [61], [62]. In neurology, the most common indications were neurotic disorders, migraines, and neuronal pain syndromes; the effects were in part explained by functional neurophysiology but also by suggestive effects [46].

## Comparison of microbiostatic efficacy of a historical high-frequency plasma device with two modern devices in vitro

### Method

The *in vitro* model for plasma susceptibility testing was performed as previously described [3]. Three plasma sources in different modes or with different electrodes were used. First, the APPJ (INP, Greifswald, Germany) was applied in three modes, one pulsed (A) and two non-pulsed (B, C). For a detailed description, see [31], [57]. Second, a Dielectric Barrier Discharge (DBD) plasma device (CINOGY, Duderstadt, Germany) was used with two dielectric barrier electrodes differing in size (20 mm diameter electrode A, 4 mm diameter electrode B). For a detailed technical description, see [58], [59]. Third, the historical CAP device, model 0126, 2 pol. constructed 1950 (Tefra, Berlin, Germany) (Figure 1 (Fig. 1)) was used. For efficacy testing we followed the settings in our previous publication [27].

All pathogens used (*Staphylococcus epidermidis* [SE], *Staphylococcus aureus* [SA], *Candida albicans* [CA], *Escherichia coli* [EC], and *Pseudomonas aeruginosa* [PA]) were clinically wound isolates. The test strains were exposed by CAP for 0, 3, 9, 15, 30, 60, and 90 s using six plasma sources/modes (DBD A, DBD B, APPJ A, APPJ B, APPJ C, and the VW) on Columbia blood agar (Biomérieux, Nürtingen, Germany). 

The diameters of the obtained inhibition areas (IA) were measured (mean of two measurements at perpendicular to each other) to calculate the susceptibility of isolates. The results give an overview of the dose response kinetics.

### Results

**Figure 3:**
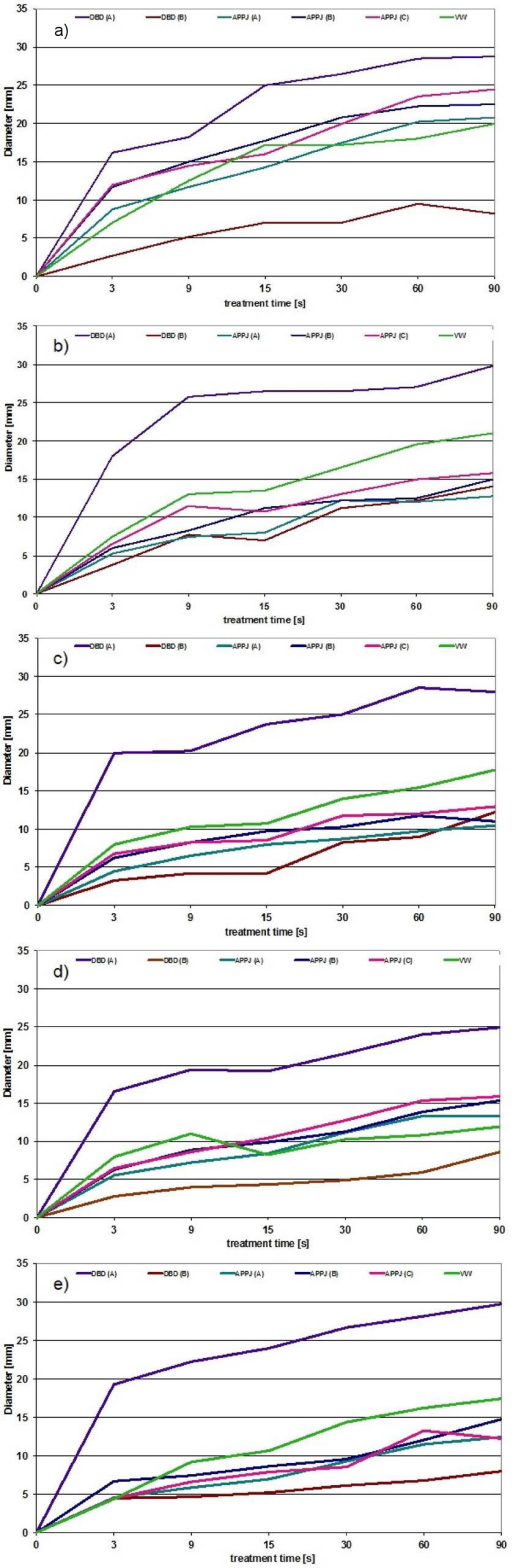
Diameter of IA after plasma treatment of selected species a) CA, b) MSSA, c) SE, d) PA, and e) EC over 3–90s with DBD (A: large, 4.5 x 4.5 mm, B: small, 2 x 2 mm electrode), pulsed and non-pulsed (2 variants) APPJ (A, B, C), and VW

The high-frequency generated plasma by the VW showed similar activity against SE, SA, CA, EC, and PA throughout the entire test phase between 3 and 90 s (Figure 3a–e (Fig. 3)). The largest diameters were recorded after DBD with the large electrode; all other treatments were similar except VW plasma (large electrode), which produced a greater diameter compared to all other treatments except DBD (large electrode), when MSSA, SE, and EC were tested (Fig. 3b, c, and e (Fig. 3)). When CA (Fig. 3a (Fig. 3)) was tested, the diameters obtained with DBD with the small electrode were markedly lower than those of all the other sources.

## Discussion

Many of the historical descriptions and claimed clinical applications are not plausible according to the standard of evidence-based medicine and systematic revision of the stated explanations is needed. However, it is possible that patients benefitted from these treatments performed in millions [63], whatever the underlying active principle may have been.

Because of the known efficacy of modern CAP, we proposed the hypothesis of similar effects by effluvia plasma discharges. To address this question, we tested basic antimicrobial properties of a representative “historical” device compared to two modern plasma sources, and can state that at least some potential beneficial clinical effect may not be purely psychosomatic. Our tests clearly demonstrated marked antimicrobial activity against all tested species *in vitro*. The effects of VW were not significantly different from those of the modern plasma devices. Thus, it is reasonable to propose a clinically relevant antibacterial effect of the VW when infected or contaminated skin was irradiated with VW plasma. 

The authors already demonstrated the in-vitro efficacy of modern CAP and historic VW generated plasma against many different wound pathogens [27], [64]. Similar data were obtained with another modern plasma source based on different technology [65]. Apart from in-vitro data, clinical studies on CAP treatment have been recently published supporting relevant efficacy against multidrug-resistant bacteria [12], [13], [14], [66]. As a result of its proven antimicrobial efficacy, CAP is currently being examined for treatment of chronic wounds [12], [67], [68] and may also be effective in hospital hygiene [67], [68], [69]. Accordingly, stimulation of wound healing supported by antiseptic activity may also be obtained with the historical VW plasma or, rather, with re-invented devices based on electromechanical techniques.

## Conclusions

Our data demonstrate *in vitro* antimicrobial efficacy of a historical CAP device and some retrograde evidence may be deducted from this, especially for different diseases that may have benefitted from antimicrobial activity.

## Notes

### Competing interests

The authors declare that they have no competing interests.

### Authorship

The authors Napp J and Daeschlein G contributed equally.
